# A Relação entre o Índice de Imuno-Inflamação Sistêmica e Isquemia com Artérias Coronárias Não Obstrutivas em Pacientes Submetidos à Angiografia Coronária

**DOI:** 10.36660/abc.20230540

**Published:** 2024-03-21

**Authors:** Muammer Karakayali, Mehmet Altunova, Turab Yakisan, Serkan Aslan, Timor Omar, Inanc Artac, Doğan Ilis, Ayca Arslan, Zihni Cagin, Yavuz Karabag, Ibrahim Rencuzogullari

**Affiliations:** 1 Kafkas University School of Medicine Department of Cardiology Kars Turquia Kafkas University School of Medicine, Department of Cardiology, Kars – Turquia; 2 Mehmet Akif Ersoy Thoracic and Cardiovascular Surgery Training Research Hospital Istanbul Turquia Mehmet Akif Ersoy Thoracic and Cardiovascular Surgery Training Research Hospital, Istanbul – Turquia; 3 Yozgat City Hospital Yozgat Turquia Yozgat City Hospital, Yozgat – Turquia

**Keywords:** Isquemia, Angiografia Coronária, Doença da Artéria Coronariana

## Abstract

**Fundamento:**

A isquemia com artéria coronária não obstrutiva (INOCA) é uma doença cardíaca isquêmica que inclui principalmente disfunção microvascular coronariana e/ou vasoespasmo coronariano epicárdico devido à disfunção vascular coronariana subjacente e pode ser observada mais comumente em pacientes do sexo feminino. O índice de inflamação imunológica sistêmica (SII, relação plaquetas × neutrófilos/linfócitos) é um novo marcador que prediz resultados clínicos adversos na doença arterial coronariana (DAC).

**Objetivo:**

Este estudo tem como objetivo investigar a relação entre INOCA e SII, um novo marcador associado à inflamação.

**Métodos:**

Um total de 424 pacientes (212 pacientes com INOCA e 212 controles normais) foram incluídos no estudo. Amostras de sangue venoso periférico foram recebidas de toda a população do estudo antes da angiografia coronária para medir o SII e outros parâmetros hematológicos. Em nosso estudo o valor de p<0,05’ foi considerado estatisticamente significativo.

**Resultados:**

O valor de corte ideal do SII para prever o INOCA foi 153,8, com sensibilidade de 44,8% e especificidade de 78,77% (Área sob a curva [AUC]: 0,651 [IC 95%: 0,603–0,696, p=0,0265]). Suas curvas ROC foram comparadas para avaliar se o SII tinha um efeito preditivo adicional valor sobre os componentes. O valor da AUC do SII foi significativamente maior do que o do linfócito (AUC: 0,607 [IC 95%: 0,559–0,654, p = 0,0273]), neutrófilos (AUC: 0,559 [IC 95%: 0,511–0,607, p = 0,028]) e plaquetas (AUC: 0,590 [IC 95%: 0,541–0,637, p = 0,0276]) em pacientes INOCA.

**Conclusões:**

Verificou-se que um nível elevado de SII estava independentemente associado à existência de INOCA. O valor do SII pode ser usado como um indicador para adicionar aos métodos tradicionais e caros comumente usados na previsão do INOCA.

**Figure:**
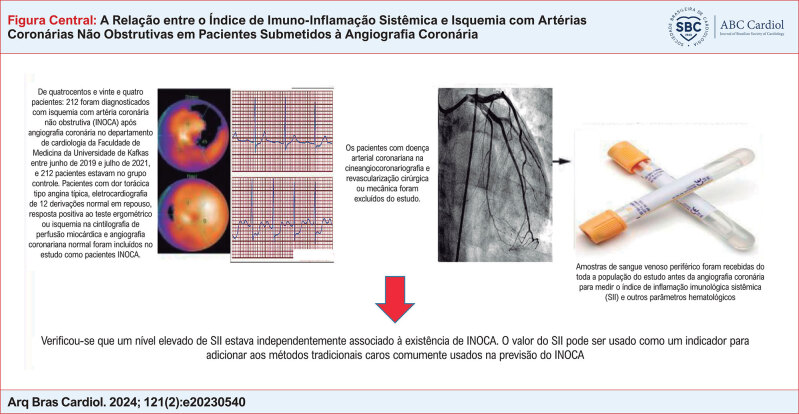


## Introdução

A maioria dos pacientes com sintomas anginosos não apresenta doença arterial coronariana (DAC) obstrutiva.^1^ Este grupo tem preponderância feminina.^2^ A disfunção vascular coronariana parece ser a causa subjacente da isquemia em até 59-89% dos pacientes chamados de ‘Isquemia sem Artérias Coronárias Obstrutivas (INOCA)’^1^ e abrange disfunção microvascular coronariana, bem como vasoespasmo coronariano epicárdico.^3^ Embora não se saiba muito sobre a patogênese da INOCA, alguns estudos afirmam que as anormalidades coronárias microcirculares e a disfunção endotelial desempenham um papel importante na patogênese da doença.^4^

O índice de imuno-inflamação sistêmica (SII) é um novo índice inflamatório que inclui três tipos de células inflamatórias que podem ser facilmente obtidos a partir de um hemograma completo e pode representar de forma mais abrangente o estado imunológico e inflamatório em pacientes (SII, relação plaquetas × neutrófilos/linfócitos).^5^ Relatos anteriores mostraram que o SII estava significativamente associado à gravidade da DAC, ao escore Syntax elevado (SxS) e aos principais eventos adversos cardiovasculares e cerebrovasculares (MACCE) em pacientes com angina de peito estável submetidos a intervenção coronária percutânea (ICP).^6 , 7^

Além disso, foi demonstrado que o SII prediz resultados clínicos intra-hospitalares e de longo prazo para pacientes idosos com infarto agudo do miocárdio (IAM) que recebem ICP, e um valor alto de SII está independentemente associado a um mau prognóstico clínico.^8^

Acredita-se que a inflamação desempenhe um papel central na etiopatogenia da INOCA; pensávamos que o SII pode estar elevado em pacientes com INOCA. Portanto, nosso objetivo foi investigar a relação do SII com os pacientes INOCA.

## Materiais e métodos

### População do estudo

Dos quatrocentos e vinte e quatro pacientes, 212 foram diagnosticados com INOCA após angiografia coronária entre junho de 2019 e julho de 2021, e 212 pacientes estavam no grupo controle. Pacientes com dor torácica típica tipo angina, eletrocardiograma de 12 derivações normal em repouso, resposta positiva ao teste ergométrico ou isquemia na cintilografia de perfusão miocárdica e angiografia coronariana normal foram incluídos no estudo como pacientes INOCA. O grupo controle incluiu pacientes com dados demográficos de idade e sexo correspondentes, ecocardiografia normal, sem evidência de isquemia no teste ergométrico ou cintilografia de perfusão miocárdica e pacientes submetidos à angiografia coronariana com suspeita de DAC, e os resultados mostraram angiografia coronariana normal.

Foram excluídos do estudo os pacientes com DAC na cineangiocoronariografia e revascularização cirúrgica ou mecânica.

Idade, sexo, hipertensão, diabetes, tabagismo e histórico familiar foram registrados como características basais. O protocolo do estudo foi aprovado pelo comitê de ética local (Comitê de Ética da Reitoria da Faculdade de Medicina - aprovação do comitê de ética 80576354-050-99/216).

### Amostras de sangue

O hemograma completo e os valores bioquímicos foram coletados retrospectivamente das amostras de sangue colhidas por via intravenosa antes da angiografia coronária. As amostras de sangue foram coletadas dos pacientes após jejum de 12 horas pela manhã. Métodos padrão foram utilizados para testes bioquímicos de rotina, incluindo perfis de glicose, ureia, creatinina e lipídios. O SII foi calculado como contagem total de plaquetas periféricas (P) × relação neutrófilos-linfócitos (N/L) (SII = relação P × N/L).^9^

### Análise angiográfica

Na angiografia coronariana (Siemens Medical Solutions, Erlangen, Alemanha), foi utilizada a técnica padrão de Judkins sem o uso de nitroglicerina. A avaliação dos angiogramas foi realizada por dois médicos experientes e cegos para o estudo. Contornos visualmente lisos e sem irregularidades de parede foram considerados normais na avaliação dos angiogramas.

### Análise estatística

No procedimento de análise dos dados foi utilizado o software SPSS versão 18.0 para Windows (SPSS Inc, Chicago, IL). O teste de Kolmogorov-Smirnov foi realizado para testar a normalidade da distribuição das variáveis contínuas. As variáveis contínuas com e sem distribuição normal foram apresentadas como média ± desvio padrão (DP) ou mediana e intervalo interquartil, respectivamente. As variáveis categóricas foram descritas como frequências absolutas e relativas. Para descobrir as diferenças nas variáveis contínuas dos grupos, foram utilizados os testes t para amostras independentes e U de Mann-Whitney de acordo com o padrão de distribuição, e o teste do qui-quadrado foi utilizado para variáveis categóricas. Para avaliar os preditores independentes do INOCA, as variáveis cujo valor de p foi <0,05 na análise de regressão logística univariada foram avaliadas por análise de regressão logística multivariada. Portanto, após a análise univariada, todas as variáveis significativas foram incluídas no modelo de regressão logística. Os resultados são apresentados como odds ratio (OR) com intervalos de confiança (IC) de 95%. Os valores de área sob a curva (AUC) obtidos a partir da análise da curva característica de operação do receptor (ROC) foram utilizados para determinar o valor preditivo poderes do SII em pacientes INOCA.

## Resultados

Um total de 424 pacientes com idade média de 56 ± 11 anos (91 [65,1%] pacientes eram mulheres) foram incluídos no estudo. Os pacientes foram divididos em dois grupos de acordo com o diagnóstico de INOCA. Os dados demográficos, bioquímicos e hematológicos basais dos pacientes de acordo com os grupos são apresentados na Tabela 1 .

**Tabela 1:** Características basais dos grupos controle e INOCA

	Grupo 0 (Grupo Controle) (n:212)		Grupo 1 (pacientes INOCA) (n:212)		Todos os pacientes		p
Anos de idade)	57	±12	54	±9	56	±11	0,158
Sexo, n (%) (feminino)	137	(64,6)	139	(65,6)	276	(65,1)	0,839
Tabagismo, n (%)	96	(45,3)	94	(44,3)	190	(44,8)	0,845
História familiar de DAC, n (%)	33	(15.6)	64	(30.2)	97	(22,9)	<0,001
Hipertensão, n (%)	90	(42,5)	89	(42,0)	179	(42,2)	0,922
Diabetes, n (%)	40	(18,9)	43	(20.3)	83	(19.6)	0,714
Hemoglobina (g/dL)	13,77	±1,54	14,91	±1,67	14h34	±1,70	<0,001
RDW	12,5	±1,9	13,5	±1,6	13	±1,8	<0,001
Plaquetas (10^3/mL)	252	±66	271	±71	261	±69	<0,001
VPM	8,72	±5,29	9,92	±1,11	9h32	3,86	<0,001
Linfócito (10^3/mL)	2,62	±0,91	2,35	±1,04	2.488	±0,985	<0,001
Neutrófilo (10^3/mL)	4,54	±1,44	4,92	±1,90	4.728	±1,695	0,034
PLR	103,38	±34,19	126,36	±47,48	114,87	42,89	<0,001
SII	169,59	(114,84-249,52)	234,86	(162,38-377,12)	200,50	(134,61-319,78)	<0,001
NLR	1,75	(1,42-2,21)	2.06	(1,55-2,71)	1,89	(1,47-2,47)	<0,001
MHR	0,00115	(0,0090-0,0175)	0,0095	(0,0072-0,0127)	0,0108	(0,0080-0,0142)	<0,001
Glicose (mg/dL)	98	±23	113	±40	106	±34	<0,001
Colesterol Total (mg/dL)	176	±41	194	±46	185	±44	<0,001
Triglicerídeo (mg/L)	127	(90-165)	144	(102-198)	137	(98-189)	0,005
LDL-C (mg/dL)	102	±38	117	±43	110	±41	<0,001
HDL-C (mg/dL)	48	±12	46	±12	47	±12	0,179
Ureia (mg/dL)	28,08	±8,097	15,04	±10,40	21h57	±11,36	<0,001
Creatina (mg/dL)	0,71	(0,62-0,83)	0,70	(0,60-0,90)	0,77	±0,45	0,056
PCRas (mg/L)	2,3	(1.1-4.4)	4,5	(3,1-8,9)	3.6	(1,6-6,9)	<0,001
FE (%)	64	±4	61	±8	62	±7	<0,001

INOCA: isquemia com artérias coronárias não obstrutivas; DAC: doença arterial coronariana, RDW: largura de distribuição de glóbulos vermelhos; VPM: volume médio de plaquetas; PLR: proporção de plaquetas para linfócitos; SII: índice de imuno-inflamação sistêmica; NLR: proporção de neutrófilos para linfócitos; MHR: Razão Monócitos/HDL-C; LDL-C: Colesterol Lipoproteico de Baixa Densidade; HDL-C: Colesterol Lipoproteico de Alta Densidade; PCRas: Proteína C Reativa de alta sensibilidade; FE: fração de ejeção.

Os pacientes com INOCA eram mais propensos a ter uma contagem mais elevada de plaquetas, relação neutrófilos-linfócitos (NLR) e valores de SII. O SII do grupo INOCA foi significativamente maior que o grupo controle. Não houve diferenças significativas em idade, sexo, tabagismo, hipertensos e diabéticos entre os grupos.

A história familiar de DAC foi estatisticamente significativamente maior nos pacientes INOCA do que no grupo controle. Dos parâmetros sanguíneos e bioquímicos: Hemoglobina (Hgb), largura de distribuição de glóbulos vermelhos (RDW), volume médio de plaquetas (VPM), relação plaquetas/linfócitos (PLR), relação monócitos/HDL-C (MHR), glicose, colesterol total (CT), triglicerídeos (TG), colesterol de lipoproteína de baixa densidade (LDL-C), creatina e proteína C reativa de alta sensibilidade (PCR-as) foram estatisticamente significativos no lado do grupo INOCA. Por outro lado, linfócitos, ureia e fração de ejeção (FE) foram maiores no lado do grupo controle.

Como resultado da análise multivariada de Hgb, VPM, SII, NLR, MHR, TG, ureia, creatinina, PCR-as, FE, LDL-C e CT, que foram considerados significativos pela análise univariada, SII, ureia, creatinina, FE e CT foram considerados preditores independentes de INOCA ( Tabela 2 ).

**Tabela 2:** Preditores significativos do INOCA na análise de regressão logística múltipla

	Univariada			Multivariada		
	OR univariada, IC 95%		p	OR multivariado, IC 95%		p
SII	1.003	(1.001-1.005)	0,011	1.004	(1.002-1.007)	0,003
Ureia	0,794	(0,761-0,828)	<0,001	0,787	(0,750-0,826)	<0,001
Creatinina	1.411	(1.205-1.752)	0,046	2.015	(1.153-3.524))	0,014
FE	0,897	(0,857-0,939)	<0,001	0,879	(0,817-0,945)	<0,001
Colesterol total	1.010	(1.005-1.014)	<0,001	1.036	(1.012-1.060)	0,003

OR: razão de chances, IC: intervalo de confiança, SII: índice de imuno-inflamação sistêmica, FE: fração de ejeção.

**Figura 1:**
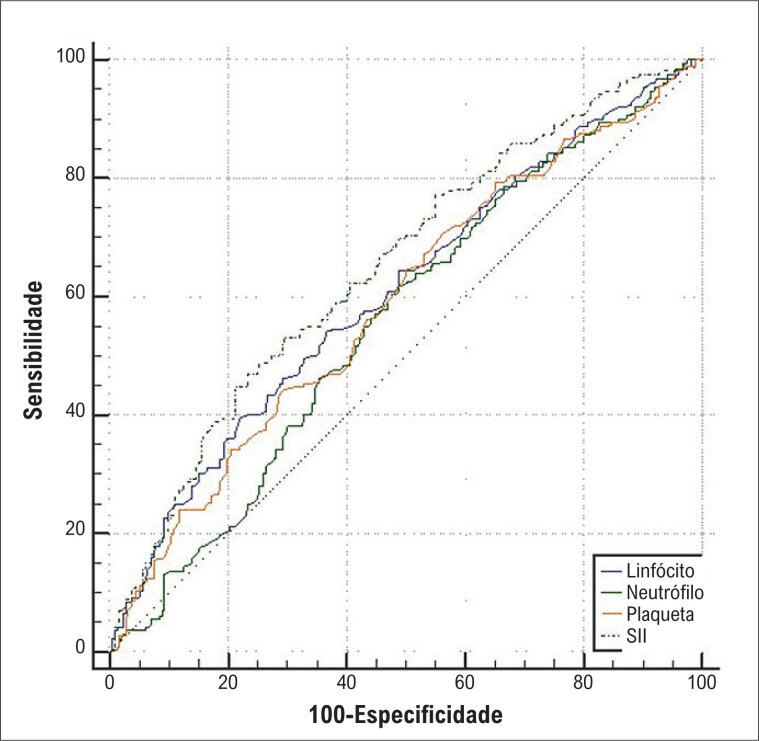
Análise da curva ROC do SII para prever INOCA.

O valor de corte ideal do SII para prever o INOCA foi 153,8, com sensibilidade de 44,8% e especificidade de 78,77% ([AUC]: 0,651 [IC 95%: 0,603–0,696, p = 0,0265]) ( Figura 1 ). Para avaliar se o SII tinha valor preditivo adicional sobre os componentes, suas curvas ROC foram comparadas. O valor da AUC do SII foi significativamente maior do que o dos linfócitos e plaquetas ( Figura 1 ) em pacientes INOCA.

## Discussão

Até onde sabemos, este é o primeiro estudo a determinar a associação entre SII e INOCA. Descobrimos que os níveis de SII foram significativamente maiores no grupo INOCA quando comparados ao grupo controle. Nosso estudo atual não tem como objetivo prever a existência de INOCA usando o índice de imuno-inflamação sistêmica (SII), antes da angiografia coronária (CAG). O diagnóstico de INOCA ainda se baseia na combinação de evidências de isquemia e exames de imagem coronarianos. Nosso objetivo neste estudo não é definir a doença sem utilizá-los. É para ajudar a identificar pacientes com maior probabilidade de ter INOCA.

Os mecanismos que contribuem para a INOCA parecem ser multifatoriais e podem funcionar isoladamente ou em combinação.^10^ Embora estes possam incluir hipertensão, estenose aórtica grave, anemia grave, enfarte do miocárdio tipo II, shunts, certos medicamentos, insuficiência cardíaca (IC) ou choque cardiogênico, angina variante de Prinzmetal (espasmo coronário), doenças do miocárdio (por exemplo, miocardite), doenças cardíacas congénitas doença coronariana, anomalias coronarianas, pontes miocárdicas e outras causas em um paciente ocasional, os mecanismos subjacentes e as estratégias apropriadas de diagnóstico e tratamento nessas situações são geralmente aparentes.

Um mecanismo proposto que contribui para a INOCA é a disfunção microvascular coronariana (DMC), definida como disfunção epicárdica, microvascular endotelial ou não endotelial que limita a perfusão miocárdica, mais frequentemente detectada como redução da reserva de fluxo coronariano (CFR).^11^ A DMC pode ocorrer na ausência de DAC obstrutiva e doença miocárdica, na doença miocárdica ou DAC obstrutiva, ou pode ser iatrogênica. Vinte e quatro disfunções vasomotoras coronarianas identificam pacientes com risco de morte cardíaca, mesmo na ausência de estenose limitante de fluxo.^12^ Existe uma distribuição de risco em toda a faixa de CFR, desde aqueles com doença obstrutiva angiográfica até aqueles com aterosclerose não obstrutiva difusa, aqueles com angiogramas de aparência normal e aqueles com apenas disfunção microvascular coronariana. Existe uma correlação limitada entre a gravidade anatômica da DAC e o comprometimento funcional, conforme refletido no CFR.^13^

O valor SII é um índice fácil de usar e econômico calculado usando as contagens de subtipos de leucócitos do teste de hemograma de rotina na admissão hospitalar. Devido aos elevados níveis de neutrófilos e plaquetas e à baixa concentração de linfócitos, um SII elevado pode estar associado ao aumento da atividade inflamatória e, portanto, levar a resultados clínicos desfavoráveis.

Estudos recentes demonstraram que o SII é um fator de risco para aterosclerose e pode ser um preditor de gravidade da lesão arterial coronariana, e um valor elevado de SII está significativamente associado ao SxS.^14^ Além disso, foi demonstrado que o SII prediz resultados clínicos intra-hospitalares e de longo prazo para pacientes idosos com IAM submetidos a ICP, e um valor alto de SII está independentemente associado a um mau prognóstico clínico.^8^

O SII é um novo e interessante marcador de inflamação e do sistema imunológico que merece ser investigado em diversas condições cardíacas. O SII é barato e não invasivo e pode ser facilmente calculado a partir de um hemograma completo. Também reflete a inflamação e a atividade do sistema imunológico em nível sistêmico.

Uma grande proporção de pacientes com INOCA apresenta disfunção microvascular. As evidências sugerem que a inflamação contribui para a disfunção microvascular que ocorre precocemente na lesão aterosclerótica. O aumento da proteína C reativa (PCR) tem sido associado ao comprometimento da função endotelial coronariana em pacientes INOCA.^15^ Em nosso estudo, os valores de PCR-as foram significativamente maiores no grupo INOCA em comparação com o grupo controle.

Em um estudo, foi demonstrado que níveis elevados de RDW estavam associados à presença de síndrome cardíaca X (SCX).^16^ Em nosso estudo, os valores de RDW foram maiores no grupo INOCA, semelhante ao CSX.

Em outro estudo realizado por Dogan, A. et al., a FCM elevada foi associada a TMC e SCX. Em nosso estudo, foi encontrada maior FCM em pacientes INOCA, semelhante à CSX.^17^

A relação entre o VPM e a gravidade angiográfica da DAC foi investigada e foi encontrada uma correlação positiva entre eles. Em um estudo realizado por Oylumlu, M. et al., os valores de VPM foram significativamente maiores nos grupos de SCX e DAC em comparação ao grupo controle.^18^ Em nosso estudo, os valores do VPM foram significativamente maiores no grupo INOCA do que no grupo controle.

Os neutrófilos suicidas podem liberar mediadores pró-oxidantes e pró-inflamatórios e causar a formação de armadilhas extracelulares de neutrófilos (NETs). Os NETs podem desencadear a formação de placas ateroscleróticas e aumentar a estabilidade do trombo.^19^ A NLR foi reconhecida como um marcador de inflamação subclínica. Na DAC, foi relatado que a RNL é um preditor independente de eventos cardiovasculares e mortalidade no IM com elevação do segmento ST.^20^ A RNL também pode ser um preditor de estenose crítica e pode estar associada tanto à gravidade quanto à morfologia da placa da doença aterosclerótica coronariana.^21^ Além da RNL, as plaquetas desempenham um papel importante na patogênese da DAC e da síndrome coronariana aguda.^22^ Agregados plaquetários intravasculares oclusivos e danos endoteliais contribuem para a etiologia da aterosclerose. As plaquetas foram vistas como um dos biomarcadores da DAC, predizendo o potencial pró-trombótico e a sensibilidade sanguínea.^23^ Foi relatado que a PLR é um preditor eficaz de aterosclerose grave.^24^ Em nosso estudo, de acordo com a literatura, a RNL e a RPL foram maiores nos pacientes INOCA do que no grupo controle.

### Limitações do estudo

Nosso estudo tem várias limitações. Este foi um estudo unicêntrico com um pequeno tamanho de amostra, e não avaliamos diretamente a velocidade do fluxo coronariano durante a provocação de acetilcolina, e o diagnóstico de espasmo microvascular não foi baseado na avaliação do fio Doppler. No entanto, isso está de acordo com os critérios aceitos.^25^ A varredura desses pacientes com um método não invasivo/angiotomografia computadorizada coronariana poderia ser considerada de acordo com a recomendação da diretriz, o que pode ser considerado uma limitação.

## Conclusões

A INOCA, um importante problema de saúde, está associada a subdiagnóstico, tratamento inadequado e mau prognóstico. Um SII mais elevado, indicando um aumento da inflamação, foi significativa e independentemente associado à presença de INOCA. Acreditamos que o valor do SII será adicionado aos métodos tradicionais e caros comumente usados na previsão do INOCA. Em conclusão, o SII, uma variável laboratorial barata e facilmente mensurável, foi um preditor independente de pacientes INOCA, mas são necessários mais estudos para apoiar plenamente esta hipótese. No entanto, estudos multicêntricos envolvendo um maior número de pacientes são necessários nesta área. É necessária investigação contínua, prospectiva e bem concebida para abordar uma série de questões não respondidas no diagnóstico e tratamento destes pacientes.
