# Supplementary material for: A Relação entre o Índice de Imuno-Inflamação Sistêmica e Isquemia com Artérias Coronárias Não Obstrutivas em Pacientes Submetidos à Angiografia Coronária
Source: Arq Bras Cardiol. 2024 Mar 21;121(2):e20230540. [Article in Portuguese] doi: 10.36660/abc.20230540 (PMC12092018; doi:10.36660/abc.20230540)
Supplement: Translation

# Translation: The Relationship between the Systemic Immune-Inflammation Index and Ischemia with Non-Obstructive Coronary Arteries in Patients Undergoing Coronary Angiography

**Authors:** Muammer Karakayali, Mehmet Altunova, Turab Yakisan, Serkan Aslan, Timor Omar, Inanc Artac, Doğan Ilis, Ayca Arslan, Zihni Cagin, Yavuz Karabag, Ibrahim Rencuzogullari

**Affiliations:** 1 Kars Turkey Kafkas University School of Medicine, Department of Cardiology, Kars – Turkey; 2 Istanbul Turkey Mehmet Akif Ersoy Thoracic and Cardiovascular Surgery Training Research Hospital, Istanbul – Turkey; 3 Yozgat Turkey Yozgat City Hospital, Yozgat – Turkey

**Keywords:** Ischemia, Coronary Angiography, Coronary Artery Disease

## Abstract

**Background:**

Ischemia with the non-obstructive coronary artery (INOCA) is an ischemic heart disease that mostly includes coronary microvascular dysfunction and/or epicardial coronary vasospasm due to underlying coronary vascular dysfunction and can be seen more commonly in female patients. The systemic immune-inflammation index (SII, platelet × neutrophil/lymphocyte ratio) is a new marker that predicts adverse clinical outcomes in coronary artery disease (CAD).

**Objective:**

This study aims to investigate the relationship between INOCA and SII, a new marker associated with inflammation.

**Methods:**

A total of 424 patients (212 patients with INOCA and 212 normal controls) were included in the study. Peripheral venous blood samples were received from the entire study population prior to coronary angiography to measure SII and other hematological parameters. In our study, the value of p<0.05’ was considered statistically significant.

**Results:**

The optimal cut-off value of SII for predicting INOCA was 153.8 with a sensitivity of 44.8% and a specificity of 78.77% (Area under the curve [AUC]: 0.651 [95% CI: 0.603–0.696, p=0.0265]). Their ROC curves were compared to assess whether SII had an additional predictive value over components. The AUC value of SII was found to be significantly higher than that of lymphocyte (AUC: 0.607 [95% CI: 0.559–0.654, p = 0.0273]), neutrophil (AUC: 0.559 [95%CI: 0.511–0.607, p=0.028]) and platelet (AUC: 0.590 [95% CI: 0.541–0.637, p = 0.0276]) in INOCA patients.

**Conclusions:**

A high SII level was found to be independently associated with the existence of INOCA. The SII value can be used as an indicator to add to the traditional expensive methods commonly used in INOCA prediction.

## Introduction

The majority of patients with anginal symptoms have no obstructive coronary artery disease (CAD).^1^This group has a female preponderance.^2^Coronary vascular dysfunction appears to be the underlying cause of ischemia in as much as 59–89% of these so-called ‘Ischaemia with No Obstructive Coronary Arteries (INOCA)’ patients^1^and encompasses coronary microvascular dysfunction (CMD) as well as epicardial coronary vasospasm.^3^Although not much is known about the pathogenesis of INOCA, certain studies asserted that micro-circular coronary abnormalities and endothelial dysfunction play an important role in the pathogenesis of the disease.^4^

Systemic immune-inflammation index (SII) is a new inflammatory index that includes 3 inflammatory cell types that can be easily obtained from a complete blood count and can more comprehensively represent the immune and inflammatory status in patients (SII, platelet × neutrophil/lymphocyte ratio).^5^Previous reports showed that SII was significantly associated with CAD severity, elevated Syntax score (SxS), and major adverse cardiovascular and cerebrovascular events (MACCE) in patients with stable angina pectoris undergoing percutaneous coronary intervention (PCI).^6 , 7^

Further, SII has been shown to predict in-hospital and long-term clinical outcomes for elderly acute myocardial infarction (AMI) patients who receive PCI, and a high SII value is independently associated with poor clinical prognosis.^8^

Inflammation is thought to play a central role in the etiopathogenesis of INOCA; we had thought that SII might be elevated in patients with INOCA. Therefore, we aimed to investigate the relationship of SII to INOCA patients.

## Materials and methods

### Study population

Of four hundred and twenty-four patients, 212 were diagnosed with INOCA after coronary angiography between June 2019 and July 2021, and 212 patients were in the control group. Patients with typical angina-like chest pain, normal 12-lead electrocardiography at rest, a positive response to the exercise test or ischemia on myocardial perfusion scintigraphy, and normal coronary angiography were included in the study as INOCA patients. The control group included patients with matching age and sex demographics, normal echocardiography, no evidence of ischemia at treadmill exercise test or myocardial perfusion scintigraphy, and patients who underwent coronary angiography with suspected CAD, and the results showed normal coronary angiography.

The patients with CAD at coronary angiography and surgical or mechanical revascularization were excluded from the study.

Age, sex, hypertension, diabetes, smoking, and family history were recorded as baseline characteristics. The study protocol was approved by the local ethics committee (Ethics Committee of the Dean of the Faculty of Medicine - numbered ethics committee approval numbered 80576354-050-99/216).

### Blood samples

Complete blood count and biochemical values were gathered retrospectively from the blood samples taken intravenously before the coronary angiography. The blood samples were collected from patients after 12 hours of fasting in the morning. Standard methods were used for routine biochemical tests, including glucose, urea, creatinine, and lipid profiles. SII was calculated as total peripheral platelets count (P) × neutrophil-to-lymphocyte ratio (N/L) (SII = P × N/L ratio).^9^

### Angiographic analysis

In coronary angiography (Siemens Medical Solutions, Erlangen, Germany), the standard Judkins technique was used without the use of nitroglycerin. The evaluation of the angiograms was conducted by two experienced physicians who were blinded to the study. Visually smooth contours with no wall irregularities were considered normal in the evaluation of angiograms.

### Statistical analysis

In the data analysis procedure, the SPSS software version 18.0 for Windows (SPSS Inc, Chicago, IL) was used. The Kolmogorov–Smirnov test was conducted to test the normality of the distribution of continuous variables. Continuous variables with and without normal distribution were presented as mean ± standard deviation (SD) or median and interquartile range, respectively. Categorical variables were described as absolute and relative frequencies. In order to figure out the differences in continuous variables of groups, independent sample t and Mann–Whitney U-tests were used according to the distribution pattern, and the chi-square test was used for categorical variables. In order to evaluate the independent predictors of INOCA, variables whose p-value was <.05 in the univariate logistic regression analysis were assessed by multivariate logistic regression analysis. Therefore, after the univariate analysis, all significant variables were included in the logistic regression model. The results are shown as odds ratio (OR) with 95% confidence intervals (CIs). The area under the curve (AUC) values obtained from the receiver operating characteristic (ROC) curve analysis were used to determine the predictive powers of SII in INOCA patients.

## Results

A total of 424 patients with an average age of 56 ±11 years (91 [65.1%] patients were female) were included in the study. The patients were divided into two groups according to the diagnosis of INOCA. The baseline demographic, biochemical, and hematological data of the patients according to the groups are presented in Table 1 .

**Table 1:** Baseline characteristics of control and INOCA groups

	Group 0 (Control Group) (n:212)		Group 1 (INOCA Patients) (n:212)		All Patients		p
Age (years)	57	±12	54	±9	56	±11	0.158
Sex, n (%) (Female)	137	(64.6)	139	(65.6)	276	(65.1)	0.839
Smoking, n (%)	96	(45.3)	94	(44.3)	190	(44.8)	0.845
Family CAD history, n (%)	33	(15.6)	64	(30.2)	97	(22.9)	<0.001
Hypertension, n (%)	90	(42.5)	89	(42.0)	179	(42.2)	0.922
Diabetes, n (%)	40	(18.9)	43	(20.3)	83	(19.6)	0.714
Hemoglobin (g/dL)	13.77	±1.54	14.91	±1.67	14h34	±1.70	<0.001
RDW	12.5	±1.9	13.5	±1.6	13	±1.8	<0.001
Platelet (10^3/mL)	252	±66	271	±71	261	±69	<0.001
MPV	8.72	±5.29	9.92	±1.11	9h32	3.86	<0.001
Lymphocyte (10^3/mL)	2.62	±0.91	2.35	±1.04	2.488	±0.985	<0.001
Neutrophil (10^3/mL)	4.54	±1.44	4.92	±1.90	4.728	±1.695	0.034
PLR	103.38	±34.19	126.36	±47.48	114.87	42.89	<0.001
SII	169.59	(114.84-249.52)	234.86	(162.38-377.12)	200.50	(134.61-319.78)	<0.001
NLR	1.75	(1.42-2.21)	2.06	(1.55-2.71)	1.89	(1.47-2.47)	<0.001
MHR	0.00115	(0.0090-0.0175)	0.0095	(0.0072-0.0127)	0.0108	(0.0080-0.0142)	<0.001
Glucose (mg/dL)	98	±23	113	±40	106	±34	<0.001
Total Cholesterol (mg/dL)	176	±41	194	±46	185	±44	<0.001
Triglyceride (mg/L)	127	(90-165)	144	(102-198)	137	(98-189)	0.005
LDL-C (mg/dL)	102	±38	117	±43	110	±41	<0.001
HDL-C (mg/dL)	48	±12	46	±12	47	±12	0.179
Urea (mg/dL)	28.08	±8.097	15.04	±10.40	21h57	±11.36	<0.001
Creatine (mg/dL)	0.71	(0.62-0.83)	0.70	(0.60-0.90)	0.77	±0.45	0.056
hs-CRP (mg/L)	2.3	(1.1-4.4)	4.5	(3.1-8.9)	3.6	(1.6-6.9)	<0.001
EF (%)	64	±4	61	±8	62	±7	<0.001

INOCA: ischaemia with Non-obstructive Coronary Arteries; CAD: coronary artery disease; RDW: red blood cell distribution width; MPV: mean platelet volume; PLR: platelet to lymphocyte ratio; SII: systemic immune-inflammation index; NLR: neutrophil to lymphocyte ratio; MHR: monocyte/HDL-C ratio; LDL-C: Low-Density Lipoprotein cholesterol; HDL-C: high-density lipoprotein cholesterol; hsCRP: high sensitive C-Reactive Protein; EF: ejection fraction.

The patients with INOCA were more likely to have a higher count of platelet, neutrophil-to-lymphocyte ratio (NLR), and SII values. SII of the INOCA group was significantly higher than the control group. There were no significant differences in age, gender, smoking status, hypertensive and diabetic patients between the groups.

Family CAD history was statistically significantly higher in INOCA patients than in the control group. From blood and biochemistry parameters: Hemoglobin (Hgb), red blood cell distribution width (RDW), mean platelet volume (MPV), platelet to lymphocyte ratio (PLR), monocyte/HDL-C ratio (MHR), glucose, total cholesterol (TC), triglyceride (TG), low-density lipoprotein cholesterol (LDL-C), creatine, high sensitive C-reactive protein (hs-CRP) were found to be statistically significant on the side of the INOCA group. On the other hand, lymphocyte, urea, and ejection fraction (EF) were found to be higher on the side of the control group.

As a result of multivariate analysis from Hgb, MPV, SII, NLR, MHR, TG, urea, creatinine, hs-CRP, EF, LDL-C, and TC, which were found to be significant by univariate analysis, SII, urea, creatinine, EF, TC were found to be independent predictors of INOCA ( Table 2 ).

**Table 2:** Significant predictors of INOCA in multiple logistic regression analysis

	Univariate			Multivariate		
	Univariate OR, 95% CI		p	Multivariate OR, 95% CI		p
SII	1.003	(1.001-1.005)	0.011	1.004	(1.002-1.007)	0.003
Urea	0.794	(0.761-0.828)	<0.001	0.787	(0.750-0.826)	<0.001
Creatinine	1.411	(1.205-1.752)	0.046	2.015	(1.153-3.524))	0.014
EF	0.897	(0.857-0.939)	<0.001	0.879	(0.817-0.945)	<0.001
Total Cholesterol	1.010	(1.005-1.014)	<0.001	1.036	(1.012-1.060)	0.003

OR: odds ratio; CI: confidence interval; SII: systemic immune-inflammation index; EF: ejection fraction

The optimal cut-off value of SII for predicting INOCA was 153.8 with a sensitivity of 44.8% and a specificity of 78.77% ([AUC]: 0.651 [95% CI: 0.603–0.696, p = 0.0265]) ( Figure 1 ). Their ROC curves were compared to assess whether SII had an additional predictive value over components, their ROC curves were compare. The AUC value of SII was found to be significantly higher than that of lymphocytes and platelets ( Figure 1 ) in INOCA patients.

**Figure 1:** ROC curve analysis of SII to predict INOCA.

## Discussion

To our knowledge, this is the first study to determine the association between SII and INOCA. We have found that SII levels were significantly higher in the INOCA group when compared to the control group. Our current study does not aim to predict INOCA existence using the systemic immune-inflammation index (SII), prior to coronary angiography. The diagnosis of INOCA is still based on a combination of ischemia evidence and coronary imaging. Our aim in this study is not to define the disease without using them. It is to help identify patients more likely to have INOCA.

The mechanisms contributing to INOCA appear to be multifactorial and may work alone or in combination.^10^Although these may include hypertension, severe aortic stenosis, severe anemia, type II MI, shunts, certain drugs, heart failure (HF) or cardiogenic shock, Prinzmetal variant angina (coronary spasm), myocardial diseases (e.g., myocarditis), congenital heart disease, coronary anomalies, myocardial bridging, and other causes in an occasional patient, underlying mechanisms and appropriate diagnostic and management strategies in these settings are usually apparent.

One proposed mechanism contributing to INOCA is CMD, defined as epicardial, microvascular endothelial, or nonendothelial dysfunction that limits myocardial perfusion, most often detected as reduced coronary flow reserve (CFR).^11^CMD may occur in the absence of obstructive CAD and myocardial disease, in myocardial disease or obstructive CAD, or may be iatrogenic. Twenty-four coronary vasomotor dysfunction identifies patients at risk of cardiac death, even in the absence of flow-limiting stenosis.^12^There is a distribution of risk across the CFR range from those with angiographic obstructive disease to those with diffuse non-obstructive atherosclerosis to those with normal-appearing angiograms to those with only CMD. There is a limited correlation between anatomic CAD severity and functional impairment, as reflected in the CFR.^13^

The SII value is an easy-to-use and cost-effective index calculated using the counts of WBC subtypes from the routine hemogram test at hospital admission. Due to high neutrophil and platelet levels and low lymphocyte concentration, a high SII may be associated with increased inflammatory activity and, therefore, lead to poor clinical outcomes.

Recent studies have shown that SII is a risk factor for atherosclerosis and may be a predictor for coronary artery lesion severity, and a high SII value is significantly associated with SxS.^14^Further, SII has been shown to predict in-hospital and long-term clinical outcomes for elderly AMI patients undergoing PCI, and a high SII value is independently associated with poor clinical prognosis.^8^

SII is a new and interesting marker of inflammation and the immune system that deserves to be investigated in various cardiac conditions. SII is inexpensive and noninvasive and can be easily calculated from a complete blood count test. It also reflects both inflammation and immune system activity at the systemic level.

A large proportion of INOCA patients have microvascular dysfunction. Evidence suggests that inflammation contributes to microvascular dysfunction that occurs early in the atherosclerotic lesion. Increased C-reactive protein (CRP) has been associated with impaired coronary endothelial function in INOCA patients.^15^In our study, hs-CRP values were found to be significantly higher in the INOCA group compared with the control group.

In one study, it was shown that high RDW levels were associated with the presence of cardiac syndrome X (CSX).^16^In our study, RDW values were found to be higher in the INOCA group, similar to CSX.

In another study by Dogan, A. et al., high MHR was associated with CMD and CSX. In our study, higher MHR was found in INOCA patients, similar to CSX.^17^

The relationship between MPV and angiographic severity of CAD was investigated, and a positive correlation between them was found. In a study by Oylumlu, M. et al., MPV values were found to be significantly higher in the CSX and CAD groups compared to the control group.^18^In our study, MPV values were found to be significantly higher in the INOCA group than in the control group.

Suicidal neutrophils can release pro-oxidant and proinflammatory mediators and cause the formation of neutrophil extracellular traps (NETs). NETs can trigger atherosclerotic plaque formation and increase thrombus stability.^19^NLR has been recognized as a marker of subclinical inflammation. In CAD, NLR has been reported to be an independent predictor of cardiovascular events and mortality in ST-segment elevation MI.^20^NLR may also be a predictor of critical stenosis and may be associated with both the severity and plaque morphology of coronary atherosclerotic disease.^21^To NLR in addition, platelets play an important role in the pathogenesis of CAD and acute coronary syndrome.^22^Occlusive intravascular platelet aggregates and endothelial damage contribute to the etiology of atherosclerosis. Platelets were seen as one of the biomarkers of CAD, predicting prothrombotic potential and blood sensitivity.^23^PLR has been reported to be an effective predictor for severe atherosclerosis.^24^In our study, in accordance with the literature, NLR and PLR were found to be higher in INOCA patients than in the control group.

### Limitations of the study

Our study has several limitations. This was a single-center study with a small sample size, and we did not directly assess coronary flow velocity during acetylcholine provocation, and the diagnosis of microvascular spasm was not based on Doppler wire assessment. However, this is in line with the accepted criteria.^25^Scanning these patients with a noninvasive method/coronary angio computerized tomography could be considered according to the guideline recommendation, this can be considered as a limitation.

## Conclusions

INOCA, an important health problem, is associated with underdiagnosis, inadequate treatment, and poor prognosis. Higher SII indicating an increased inflammation was significantly and independently associated with the presence of INOCA. We think that the value of SII will add to the traditional expensive methods commonly used in INOCA prediction. In conclusion, SII, an inexpensive and easily measurable laboratory variable, was an independent predictor of INOCA patients, but further studies are needed to support this hypothesis fully. However, multicentre studies involving larger numbers of patients are needed in this field. Prospective, well-designed ongoing research is needed to address a number of unanswered questions in the diagnosis and management of these patients.
